# Turmeric Bioactive Compounds Alleviate Spinal Nerve Ligation-Induced Neuropathic Pain by Suppressing Glial Activation and Improving Mitochondrial Function in Spinal Cord and Amygdala

**DOI:** 10.3390/nu15204403

**Published:** 2023-10-17

**Authors:** Julianna M. Santos, Rui Wang, Viren Bhakta, Zarek Driver, Yakhnitsa Vadim, Takaki Kiritoshi, Guangchen Ji, Volker Neugebauer, Chwan-Li Shen

**Affiliations:** 1 Department of Pathology, Texas Tech University Health Sciences Center, Lubbock, TX 79430, USA; julianna-maria.santos@ttuhsc.edu (J.M.S.); rui.wang1@ufl.edu (R.W.); 2 Department of Biochemistry, Texas Tech University, Lubbock, TX 79409, USAzdriver@ttu.edu (Z.D.); 3 Department of Pharmacology and Neurosciences, Texas Tech University Health Science Center, Lubbock, TX 79430, USA; vadim.yakhnitsa@ttuhsc.edu (Y.V.); takaki.kiritoshi@ttuhsc.edu (T.K.); guangchen.ji@ttuhsc.edu (G.J.); volker.neugebauer@ttuhsc.edu (V.N.); 4 Center of Excellence for Translational Neuroscience and Therapeutics, Texas Tech University Health Sciences Center, Lubbock, TX 79430, USA; 5 Garrison Institute on Aging, Texas Tech University Health Sciences Center, Lubbock, TX 79430, USA; 6 Center of Excellence for Integrative Health, Texas Tech University Health Sciences Center, Lubbock, TX 79430, USA

**Keywords:** curcumin, pain, neuroinflammation, brain, animal

## Abstract

This study examined the effects of turmeric bioactive compounds, curcumin C3 complex® (CUR) and bisdemethoxycurcumin (BDMC), on mechanical hypersensitivity and the gene expression of markers for glial activation, mitochondrial function, and oxidative stress in the spinal cord and amygdala of rats with neuropathic pain (NP). Twenty-four animals were randomly assigned to four groups: sham, spinal nerve ligation (SNL, an NP model), SNL+100 mg CUR/kg BW p.o., and SNL+50 mg BDMC/kg BW p.o. for 4 weeks. Mechanical hypersensitivity was assessed by the von Frey test (VFT) weekly. The lumbosacral section of the spinal cord and the right amygdala (central nucleus) were collected to determine the mRNA expression of genes (IBA-1, CD11b, GFAP, MFN1, DRP1, FIS1, PGC1α, PINK, Complex I, TLR4, and SOD1) utilizing qRT-PCR. Increased mechanical hypersensitivity and increased gene expression of markers for microglial activation (IBA-1 in the amygdala and CD11b in the spinal cord), astrocyte activation (GFAP in the spinal cord), mitochondrial dysfunction (PGC1α in the amygdala), and oxidative stress (TLR4 in the spinal cord and amygdala) were found in untreated SNL rats. Oral administration of CUR and BDMC significantly decreased mechanical hypersensitivity. CUR decreased CD11b and GFAP gene expression in the spinal cord. BDMC decreased IBA-1 in the spinal cord and amygdala as well as CD11b and GFAP in the spinal cord. Both CUR and BDMC reduced PGC1α gene expression in the amygdala, PINK1 gene expression in the spinal cord, and TLR4 in the spinal cord and amygdala, while they increased Complex I and SOD1 gene expression in the spinal cord. CUR and BDMC administration decreased mechanical hypersensitivity in NP by mitigating glial activation, oxidative stress, and mitochondrial dysfunction.

## 1. Introduction

Chronic pain is a major public health problem, and about a fifth of the general population is affected by chronic pain in the USA [1]. Currently, available treatments (i.e., opioids and non-steroidal anti-inflammatory drugs) for chronic pain are often inadequate or lack efficacy and have severe side effects [2]. A better understanding of chronic pain mechanisms is required for novel and improved therapeutic strategies.

Nerve-injury-induced neuropathic pain (NP) is a chronic pain condition that involves neuroinflammation and neuroplastic changes in the peripheral and central neurons associated with sensitization and hyperexcitability [3]. At the molecular level, excessive oxidative stress results from increased reactive oxygen species (ROS), leading to mitochondrial damage during the development of NP. Low-grade inflammation in the central nervous system (CNS), glial dysfunction, and subsequent imbalances in neuron–glial interactions have emerged as important contributors to chronic NP, causing central sensitization in pain pathways and increased responsiveness to painful stimuli [4]. Thus, it is important to reduce excessive oxidative stress, proinflammatory cytokine release, and microglial activation in order to decrease CNS neuroinflammation in the management of NP [5]. Mitochondrial-derived damage-associated molecular factors amplify neuroinflammation, contributing to NP progression [6]. Mitochondrial dysfunction has been implicated in sensory processing and NP. Increased mitochondrial ROS production or mitochondrial Ca^2+^ concentration can trigger nucleotide-binding oligomerization domain-like receptor pyrin domain containing 3 (NLRP3) inflammasome activation and/or binding of Parkin and PTEN-induced kinase 1 (PINK1) to mitochondria, resulting in the elimination of damaged mitochondria via mitophagy [7]. ROS-damaged mitochondrial DNA induces pro-inflammatory IL-1β production through NLRP3 inflammasome activation in NP [8]. Chronic inflammation and excessive oxidative stress reduce mitochondrial transport and impair mitochondrial quality control mechanisms, such as mitophagy, mitochondrial biogenesis, fusion, and fission. Improving impaired mitochondrial functions in sensory neurons has been shown to reduce hyperalgesia in pre-clinical models of NP [7]. Mitochondria-targeting antioxidants, such as dietary polyphenols, have therapeutic effects on a myriad of pathological conditions, notably NP. In the past decade, the beneficial effects of bioactive compounds, including dietary polyphenols, in NP have gained much attention due to their capability to interact directly or indirectly with peripheral and central nervous system signaling via their anti-inflammatory and antioxidant properties.

The ground rhizome of herbal *Curcuma longa* (turmeric) and its constituent, curcumin (a polyphenol), have long been used therapeutically for their anti-inflammatory, antioxidant, and anti-nociceptive effects [9]. The anti-nociceptive effects of bioactive turmeric compounds, especially curcumin, on NP-related behaviors in a number of animal models have been reviewed recently [10]. Turmeric compounds’ anti-nociceptive abilities are associated with opioid receptor systems, anti-inflammatory effects, the suppression of glial activation, and synaptic transmission modulation. However, no studies have evaluated how turmeric extract and its bioactive compounds affect glial activation, mitochondrial function (mitophagy, mitochondrial biogenesis, fission, and fusion), and oxidative stress in the spinal cord and the amygdala of animals with NP.

The spinal cord is the interface between the peripheral and central nervous system and transmits information to the brain. The amygdala significantly influences the emotional-affective aspects of pain and pain modulation [11]. Therefore, this study was designed to investigate the effect of turmeric extract, curcumin C3 complex^®^ (CUR, containing 97% curcuminoids) and bisdemethoxycurcumin (BDMC, a bioactive curcuminoid compound found in turmeric) on mechanical hypersensitivity, glial activation, mitochondrial function, and oxidative stress in male rats in a spinal nerve ligation (SNL) model of NP. We hypothesized that both CUR and BDMC administration would decrease mechanical hypersensitivity, suppress glial activation, and improve mitochondrial function in the spinal cord and amygdala of SNL animals. The effects of CUR and BDMC would be mediated, at least in part, by the suppression of neuroinflammation and oxidative stress, as well as the elevation of antioxidant and metabolic function.

## 2. Materials and Methods

### 2.1. Animals

Twenty-four male Sprague Dawley rats (150–180 g body weight) were purchased from Envigo (Cumberland, VA, USA) and housed individually under a 12 h light–dark cycle with food and water ad libitum. All procedures were approved by the Institutional Animal Care and Use Committee at Texas Tech University Health Sciences Center (IACUC # 21007). All experiments were performed in accordance with the relevant guidelines and regulations.

### 2.2. Induction of Neuropathic Pain

After 5 days of acclimatization, 6 animals were randomly assigned as sham-controls receiving sham surgery, while the remaining 18 animals underwent SNL procedures. The SNL model was used to induce NP in the left hind paw, as described in our previous work [3,12]. In brief, isoflurane was used for the induction (3%) and maintenance (2%) of anesthesia throughout the operation. After removing the L5/L6 level paraspinal muscles and underlying L6 transverse process, the L5 spinal nerve was removed from adjacent structures and securely ligated with 6–0 silk thread. The paraspinal muscles were closed with sutures, and the skin was clipped together. Sham-operated animals served as controls for the NP model, receiving the same surgical steps leading up to spinal nerve ligation. After surgery, all animals were given antibiotic treatment (1 dose of gentamycin, 8 mg/kg, subcutaneously, s.c.; VetOne, Boise, ID) and were monitored for any signs of infection or distress. Throughout the study period, the animals were monitored to reduce unnecessary stress or pain following ethical guidelines of the International Association for the Study of Pain [13].

### 2.3. Dietary Treatments

Turmeric extract contains three curcuminoids, namely curcumin (80%, relative abundance), demethoxycurcumin (~15%), and BDMC (~5%), which have different methoxy substitutions on the aromatic ring [14]. Curcumin C3 complex^®^ (CUR) is a standardized extract containing a ratio-defined mixture of three curcuminoids (curcumin, demethoxycurcumin, and BDMC) that achieved the GRAS (Generally Recognized as Safe) status. Both BDMC and demethoxycurcumin compounds stabilize curcumin and make curcumin more efficiently absorbed in the GI tract [15]. We selected to test purified BDMC and compare it with CUR in an animal NP model for this study for the following reasons: (i) BDMC has been shown to be more stable than the other two curcuminoids (curcumin and demthoxycurcumin) and (ii) BDMC possess numerous pharmacological actions, such as antioxidant [16], anti-inflammatory [17], pro-apoptotic [18], analgesic [19], and neuroprotective [20] effects. BDMC’s multi-mechanistic mode of action would enable its potential efficacy in treating several pathophysiological conditions, such as NP.

**Figure 1:**
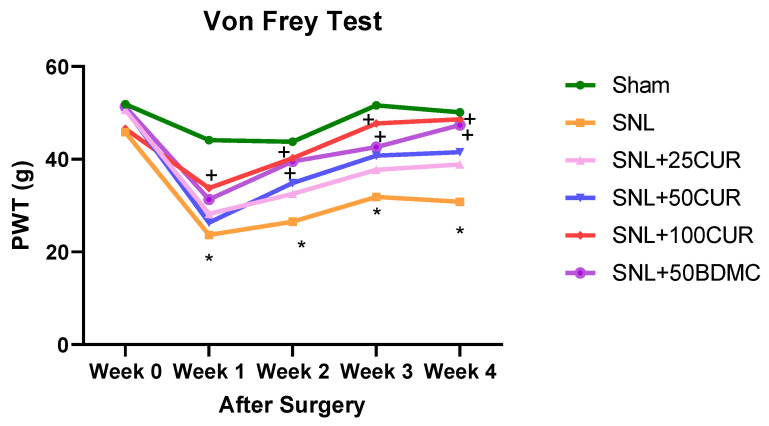
Supplementation of turmeric bioactive compounds increased mechanical thresholds in SNL rats as assessed by an electronic von Frey (VFT) aesthesiometer. Data are expressed as mean ± SEM. Six groups include Sham, SNL, SNL+25CUR (25 mg curcumin C3 complex^®^/BW daily), SNL+50CUR (50 mg curcumin C3 complex^®^/BW daily), SNL+100CUR (100 mg curcumin C3 complex^®^/BW daily), and SNL+50BDMC (50 mg bisdemethoxycurcumin/BW daily). *n* = 5–6 animals per group. At each week, data were analyzed by one-way ANOVA followed by post hoc Tukey’s test. * *p* < 0.05 SNL vs. Sham. + *p* < 0.05 SNL+100CUR vs. SNL and SNL+50BDMC vs. SNL.

Based on our pilot study using 3 different doses of CUR (25, 50, and 100 mg/kg BW, p.o.) and BDMC (50 mg/kg BW), we found there was no significant difference between CUR at 100 mg/kg BW dose and BDMC at 50 mg/kg BW p.o. in the pain mechanical hypersensitivity of SNL-treated rats (see Figure 1). Therefore, we selected CUR at 100 mg/kg BW and BDMC at 50 mg/kg BW for the present study to evaluate their effects on glial activation, mitochondrial function, and oxidative stress.

We randomly divided the 24 animals into four dietary treatment groups: sham group, SNL group, SNL+CUR at 100 mg/kg BW, p.o. (SNL+100CUR group), and SNL+BDMC at 50 mg/kg BW, p.o. (SNL+50BDMC group). All animals were given AIN-93G diet (catalog number # D10012G, Research Diet, Inc., New Brunswick, NJ, USA). For the SNL+100CUR and SNL+50BDMC groups, the animals were given CUR and BDMC, respectively, on the day of SNL surgery for 4 weeks. Both CUR and BDMC (>99% purity from turmeric extract) were a gift from Sabinsa Corporation, East Windsor, NJ. CUR has 97.34% total curcuminoids including 77.3% curcumin, 19.0% demethoxycurcumin, and 3.7% BDMC. Body weight, food intake, and water consumption were recorded weekly.

### 2.4. Pain Assessment

The mechanical withdrawal thresholds of spinal nocifensive reflexes were evaluated on the left hind paw using an Electronic von Frey Aesthesiometer (IITC Life Science, Woodland Hills, CA, USA) with a plastic tip (catalog number 76-0488, Harvard Apparatus, Holliston, MA, USA) in a dedicated testing area at baseline (week 0) and at the end of the study (week 4) after the respective treatments. The average of six measurements per subject, taken at least 30s apart, was calculated.

### 2.5. Sample Collection

At the end of the experiment, the animals were anesthetized with isoflurane before euthanization, and their blood was drawn for plasma and serum collection. The spinal cord (lumbosacral) and the amygdala (right central nucleus) were collected, immersed in liquid nitrogen, and stored at −80 °C for later mRNA expression analysis.

### 2.6. RNA Isolation and qRT-PCR

Total RNA was isolated from spinal cord and amygdala using the RNAzol RT (RN190, Molecular Research Center Inc., Cincinnati, OH, USA) and BAN (BN191, Molecular Research Center, Cincinnati, OH, USA) ratio at 1:200. Total RNA was quantified using nanodrop at 260 nm (Nanodrop one, Thermo Scientific, Waltham, MA, USA) and then reverse transcribed into cDNA using a thermal cycler (Bio-Rad S1000, Bio-Rad Laboratories, Inc., Hercules, CA, USA). qRT-PCR was performed on Quant Studio 12K Flex real-time PCR system (Life Technologies, 4470689, Carlsbad, CA, USA) for cDNA for amplification of target genes (Table 1) with Universal SYBR green supermix (Bio-rad Laboratories, Inc., 17251-24, Hercules, CA, USA). The relative quantification of the gene expression was calculated with 2−(ΔCT*1000) values normalized to respective β-actin values [21].

### 2.7. Statistical Analysis

The data are presented as the mean ± standard error of the mean (SEM). For data from von Frey tests, one-way ANOVA followed by a *posthoc* Tukey’s test was conducted at each collection time to examine group differences. mRNA gene expression data were analyzed by unpaired *t*-test for model comparison (Sham vs. SNL) and treatment comparison (SNL vs. SNL+100CUR and SNL vs. SNL+50BDMC) [21,22]. Data was analyzed with GraphPad Prism 9 (GraphPad Software, San Diego, CA, USA). Statistical significance was accepted at the level of *p* < 0.05 (*).

## 3. Results

### 3.1. Turmeric Bioactive Compounds Supplementation Mitigated Mechanical Hypersensitivity

Mechanical hypersensitivity for pain was measured by the von Frey test. Compared with the sham group, the SNL group had significantly greater mechanosensitivity starting at 1-week post-induction, which lasted throughout the study period (Figure 1).

At the end of study, among all turmeric bioactive compounds, only CUR at 100 mg/kg BW and BDMC at 50 mg/kg BW had significantly decreased hypersensitivity relative to the untreated SNL group. The pain-reduction impact of 100CUR and 50BDMC started as early as 2 weeks post induction, which is 1 week after starting CUR or BDMC, and the effect persisted for 4 weeks, as demonstrated by the increased mechanical thresholds.

### 3.2. Turmeric Bioactive Compounds Supplementation Altered mRNA Expression of Microglial and Astrocyte Activation

**Figure 2:**
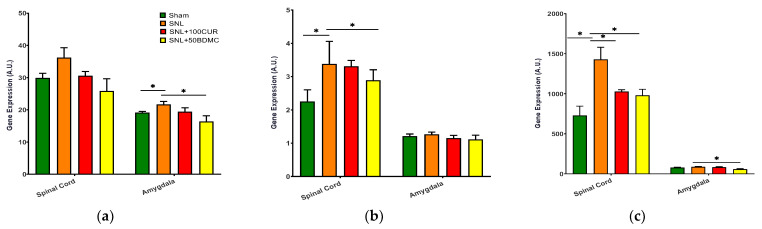
Effect of turmeric bioactive compounds supplementation on IBA-1 (**a**), CD11b (**b**), and GFAP (**c**) mRNA expression in the spinal cord and amygdala of SNL rats. Data are expressed as mean ± SEM. Four groups included Sham, SNL, SNL+SNL+100CUR (100 mg curcumin C3 complex^®^/BW daily), and SNL+50BDMC (50 mg bisdemethoxycurcumin/BW daily). *n* = 5–6 animals per group. Data were analyzed by unpaired *t*-test Sham vs. SNL, SNL vs. SNL+100CUR, and SNL vs. SNL+50BDMC. * *p* < 0.05.

We examined the effects turmeric bioactive compounds on gene expression of microglial activation [IBA-1 (Figure 2a) and CD11b (Figure 2b)] and astrocyte activation [GFAP (Figure 2c)] in the spinal cord and amygdala. IBA-1 gene expression increased in the spinal cord (although not statistically significant) and amygdala of SNL rats, and was significantly decreased by 50BDMC, whereas 100CUR showed a non-significant trend (Figure 2a). CD11b gene expression increased significantly in the spinal cord, but not the amygdala, of SNL rats, and was decreased by both 100CUR and 50DMC compounds (Figure 2b). GFAP gene expression increased in the spinal cord, but not the amygdala, of SNL rats, and was decreased by 100CUR and 50BDMC in the spinal cord of rats, whereas 50BDMC also decreased GFAP gene expression in the amygdala (Figure 2c).

### 3.3. Turmeric Bioactive Compound Supplementation Altered mRNA Expression of Mitochondrial Fusion, Fission, and Biogenesis Markers

**Figure 3:**
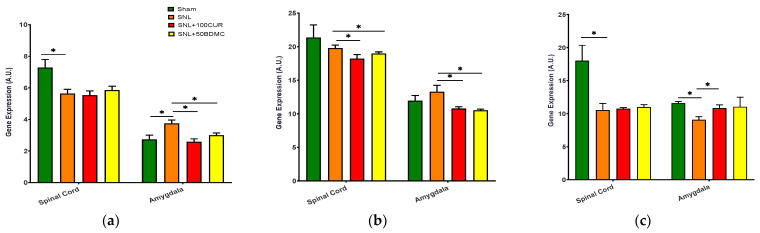
Effect of turmeric bioactive compounds supplementation on MFN1 (**a**), DRP1 (**b**), and FIS1 (**c**) gene expression in the spinal cord and amygdala of SNL rats. Data are expressed as mean ± SEM. Four groups included Sham, SNL, SNL+100CUR (100 mg curcumin C3 complex^®^/BW daily), and SNL+50BDMC (50 mg bisdemethoxycurcumin/BW daily). *n* = 5–6 animals per group. Data were analyzed by unpaired *t*-test Sham vs. SNL, SNL vs. SNL+100CUR, and SNL vs. SNL+50BDMC. * *p* < 0.05.

We investigated the effects turmeric bioactive compounds supplementation on MFN1 (Figure 3a) gene expression for mitochondrial fusion, as well as DPR1 (Figure 3b) and FIS1 (Figure 3c) gene expression for mitochondrial fission in the spinal cord and amygdala of SNL rats. Compared to the Sham group, the SNL group had elevated MFN1 gene expression in the amygdala but decreased levels in the spinal cord of SNL rats. Both 100CUR and 50BDMC significantly suppressed MFN1 gene expression in the amygdala, but not the spinal cord (Figure 3a). There was no significant difference in DRP1 gene expression between the Sham and the SNL group in the spinal cord and amygdala. Relative to the untreated SNL group, both SNL+100CUR and SNL+50BDMC groups had significantly lower DRP1 gene expression in both tissues (Figure 3b). FIS1 gene expression was significantly decreased in the spinal cord and amygdala. The SNL+100CUR group showed increased FIS1 gene expression in the amygdala only. BDMC had no significant effect on FIS1 gene expression (Figure 3c).

**Figure 4:**
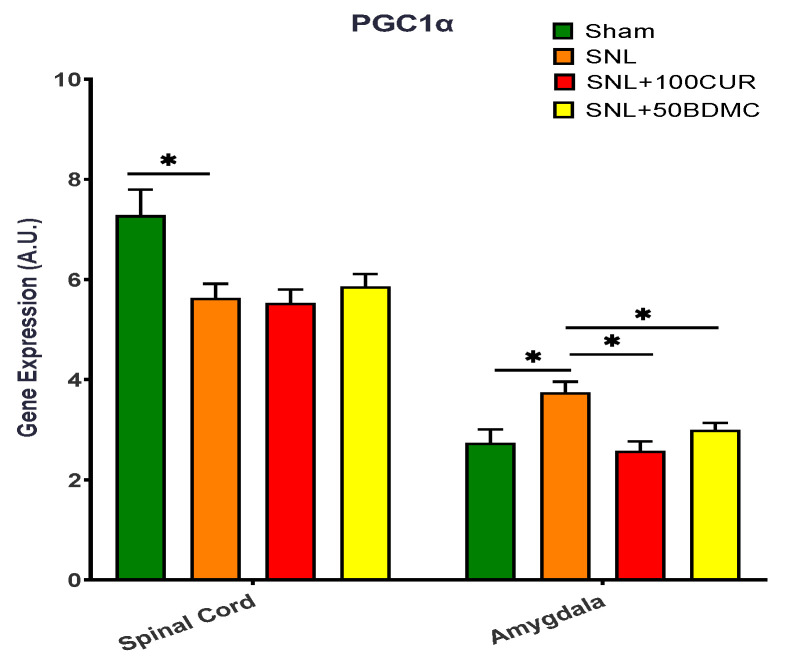
Effect of turmeric bioactive compounds supplementation on PGC1α gene expression in the spinal cord and amygdala of SNL rats. Data are expressed as mean ± SEM. Four groups included Sham, SNL, SNL+100CUR (100 mg curcumin C3 complex^®^/BW daily), and SNL+50BDMC (50 mg bisdemethoxycurcumin/BW daily). *n* = 5–6 animals per group. Data were analyzed by unpaired *t*-test Sham vs. SNL, SNL vs. SNL+100CUR, and SNL vs. SNL+50BDMC. * *p* < 0.05.

The effects of turmeric bioactive compounds supplementation were also studied on PGC1α gene expression in the spinal cord and amygdala of SNL rats (Figure 4). Relative to the Sham group, the SNL group had decreased PGC1α gene expression in the spinal cord, but increased PGC1α gene expression in the amygdala. Both 100CUR and 50BDMC supplementation had no significant effect on PGC1α gene expression in the spinal cord of SNL rats. On the other hand, both 100CUR and 50BDMC significantly decreased PGC1α gene expression in the amygdala of SNL.

### 3.4. Turmeric Bioactive Compounds Supplementation Suppressed mRNA Expression of Mitochondrial Autophagy and Oxidative Stress Markers

**Figure 5:**
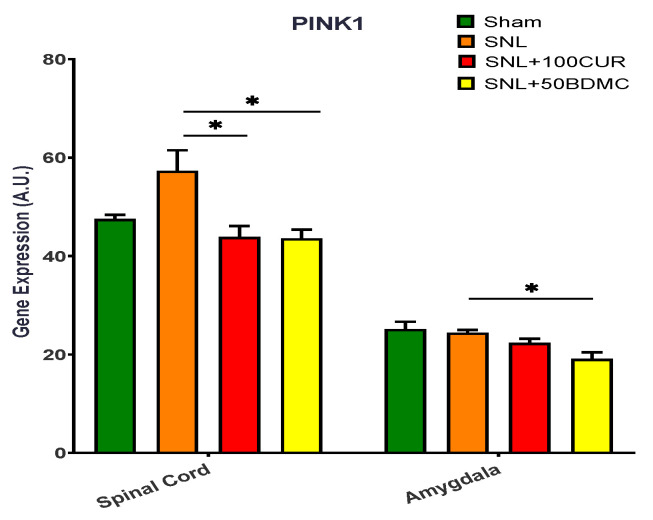
Effects of turmeric bioactive compounds supplementation on gene expression of PINK1, a mitochondrial kinase, in the spinal cord and amygdala of SNL rats. Data are expressed as mean ± SEM. Four groups included Sham, SNL, SNL+100CUR (100 mg curcumin C3 complex^®^/BW daily), and SNL+50BDMC (50 mg bisdemethoxycurcumin/BW daily). *n* = 5–6 animals per group. Data were analyzed by unpaired *t*-test Sham vs. SNL, SNL vs. SNL+100CUR, and SNL vs. SNL+50BDMC. * *p* < 0.05.

The effects of turmeric bioactive compounds supplementation were tested on PINK1 gene expression for mitochondrial autophagy in SNL rats (Figure 5). PINK1 gene expression was significantly increased in the spinal cord, but not the amygdala, of SNL rats. Interestingly, both 100CUR and 50BDMC supplementation significantly suppressed PINK1 gene expression in the spinal cord and amygdala of SNL rats.

**Figure 6:**
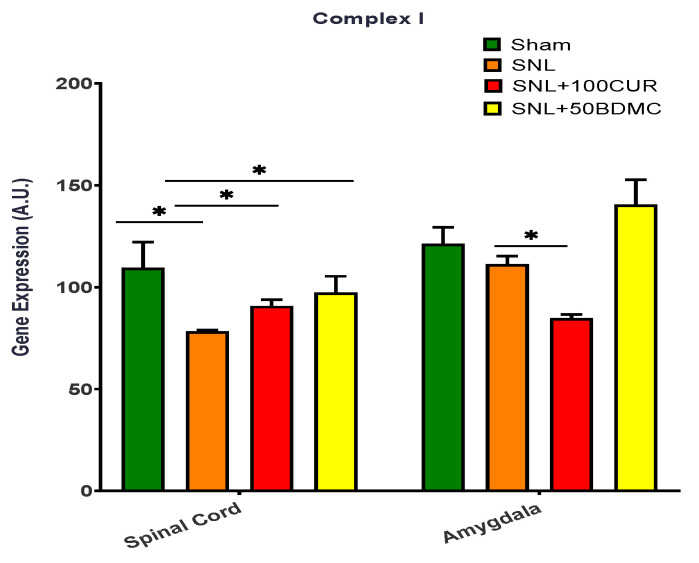
Effects of turmeric bioactive compounds supplementation on Complex I in the spinal cord and amygdala of SNL rats. Data are expressed as mean ± SEM. Four groups included Sham, SNL, SNL+100CUR (100 mg curcumin C3 complex^®^/BW daily), and SNL+50BDMC (50 mg bisdemethoxycurcumin/BW daily). *n* = 5–6 animals per group. Data were analyzed by unpaired *t*-test Sham vs. SNL, SNL vs. SNL+100CUR, and SNL vs. SNL+50BDMC. * *p* < 0.05.

The effects of turmeric bioactive compounds supplementation were tested on complex I gene expression for mitochondrial respiratory chain complexes (Figure 6). Complex I gene expression was significantly decreased in the spinal cord, but not the amygdala, of SNL rats. The 50BDMC supplementation resulted in increased Complex I gene expression in both spinal cord and amygdala of NP rats. However, the differential effects of 100CUR supplementation were observed in NP rats, namely increased Complex I gene expression in spinal cord and decreased Complex I gene expression in amygdala.

**Figure 7:**
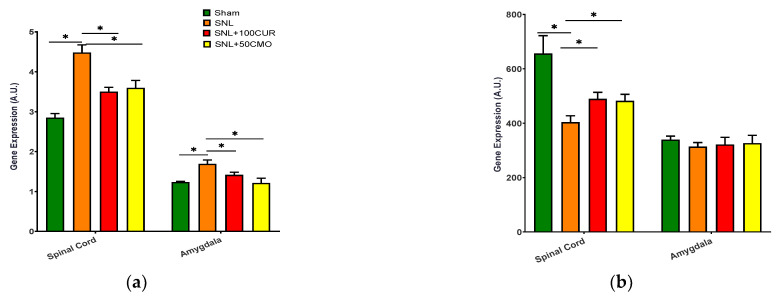
Effects of turmeric bioactive compounds supplementation on TLR4 (**a**) and SOD1 (**b**) in the spinal cord and amygdala of SNL rats. Data are expressed as mean ± SEM. Four groups included Sham, SNL, SNL+100CUR (100 mg curcumin C3 complex^®^/BW daily), and SNL+50BDMC (50 mg bisdemethoxycurcumin/BW daily). *n* = 5–6 animals per group. Data were analyzed by unpaired *t*-test Sham vs. SNL, SNL vs. SNL+100CUR, and SNL vs. SNL+50BDMC. * *p* < 0.05.

The effects of turmeric bioactive compounds supplementation were tested on TLR4 (Figure 7a) and SOD1 (Figure 7b) gene expression for mitochondrial oxidative stress. TLR4 gene expression increased significantly in the spinal cord and amygdala of SNL rats and was decreased by both CUR and BDMC supplementation (Figure 7a). Increased oxidative stress (decreased SOD1) was found in the spinal cord, but not the amygdala, of SNL rats and was significantly mitigated by CUR and BDMC supplementation (Figure 7b).

## 4. Discussion

This study is the first to analyze the effects of turmeric bioactive compounds on pain behaviors and molecular markers of glial expression, mitochondrial function, and oxidative stress in the SNL model of NP. Consistent with our previous studies, SNL rats developed sustained hypersensitivity [3,12,23,24,25,26]. The present study demonstrated the potent effects of 4-week curcumin C3 complex^®^ (CUR) and pure BDMC supplementation (first time) in mitigating mechanical hypersensitivity in male SNL rats. Such findings corroborate findings from previous studies [27,28] that used purified 100% curcumin compound in SNL animals and found inhibitory effects on mechanical allodynia.

A key novelty of the present study is the analysis of the potential mechanisms of CUR and BDMC actions by exploring their effects on glial activation, mitochondrial function, and oxidative stress in the spinal cord and amygdala of SNL rats. Our data of IBA-1, CD11b, and GFAP corroborated others for the role of glial activation in NP development [29,30,31]. The regulatory function of the CD11b-Src signaling pathways on both inflammatory and anti-inflammatory cytokines in microglia cells is a potential target in NP treatment [32]. Astrocyte reaction is more persistent than microglial activation in the spinal cord after painful injuries, and the temporally distinct purposes of microglia and astrocytes have been discussed [33]. Activation of GFAP (an astrocyte marker) plays a key role in the chronic phase of NP, while activation of IBA-1 (a microglial marker) may more closely correlate with the early phase of NP [34]. The present study shows a positive correlation between hyperalgesia, microglial activation (increased CD11b gene expression), and astrocyte activation (increased GFAP gene expression) in the spinal cord of SNL rats at the chronic stage, which is consistent with previous work on the connection between microglial and astrocyte activation and the onset and maintenance of NP [35]. The lack of statistical difference in SNL’s up-regulatory effect on the IBA-1 gene expression (usually occurring at an early stage of NP) in the spinal cord of rats may be due to the timing these aspects were examined, which was at a chronic stage of NP (after 4-week post-surgery). On the other hand, we observed IBA-1, not CD11b and GFAP, gene upregulation in NP in the amygdala, a key brain region for the modulation of emotion and pain, which agrees with Hu’s study in spared nerve injury-induced NP rats [36]. To our best knowledge, this study is the first to show that turmeric bioactive compounds (CUR containing 3 curcuminoids and purified BDMC), especially BDMC, reverted SNL-induced IBA-1, CD11b, and GFAP gene expression in specific CNS tissues (i.e., spinal cord and/or amygdala). Such inhibitory effects from curcuminoids on GFAP gene expression have been found in the spinal dorsal horn in CCI-induced NP rats [37] and in the whole brain in streptozotocin-induced NP rats using curcumin derivative J147 [38] (purified curcumin). Our results provide further evidence for the beneficial antinociceptive effects of curcumin supplementation through the mitigation of microglial and astrocyte activation in the CNS.

Excessive oxidative stress can cause NP by reducing spinal inhibitory transmission and increasing afferent excitatory transmission, forming imbalanced/disrupted mitochondrial dynamics [39] in the spinal dorsal during NP progression [40,41,42,43]. Antioxidant therapy has been shown to be effective in CCI-induced NP rats via improving mitochondrial imbalanced/disrupted dynamics, quality, and number [44] in the dorsal root ganglion, due to free radical scavenging activity [45]. In the present study, we found that relative to the Sham rats, untreated SNL rats had increased fusion (MFN1) and decreased fission (FIS1) in the spinal cord, indicating imbalanced mitochondrial dynamics. Both CUR and BDMC reduced fission (DRP1) and fusion (MFN1) in the spinal cord and amygdala of SNL rats, suggesting a lower turnover of mitochondrial dynamics in curcumin-supplemented CNS tissues. Intriguingly, in this study the decreased DRP1/FIS1 ratio in the spinal cord and amygdala of both curcuminoids-supplemented SNL groups suggests that curcuminoid supplementation favors fission over fusion, resulting in improved mitochondrial dynamics. This present study showed that both CUR and BDMC supplementation significantly decreased the expression levels of DRP1 compared to SNL animals, corroborating that curcumin decreases mitochondria fragmentation [46]. Dysfunctional mitochondria produce ROS that in turn promote inflammation and shift the mitochondrial dynamic towards fission [47,48].

PGC-1α enhances the balance between ROS production and its free radical scavenging process during neuroinflammation by regulating key antioxidant gene expression [49]. The finding that both curcuminoid supplementations (CUR and BDMC) revert the SNL-induced increase in PGC-1α gene expression in the amygdala in NP supports the previous studies [50,51]. Tetrahydro curcumin (a derivative of curcumin) had beneficial effects on mitochondrial remodeling in brain endothelial cells via the improved mitochondrial function (decreased DRP-1, MFN2, and autophagy maker LC-3) [50]. Curcumin administration increased PGC-1α gene expression in hepatic stellate cells via the activation of adenosine monophosphate-activated protein kinase pathways (acting as a cellular energy sensor) [51]. Interestingly, PGC-1α gene expression was differentially affected in the spinal cord and amygdala in the NP model, with enhanced PGC-1α gene expression in the amygdala. It is possible that compensatory effects on PGC-1α expression between the nearby spinal cord and the more remote amygdala explain the region-specific differential change.

Dysregulation of the autophagic machinery has been associated with NP mechanisms [52], including in the SNL model [53]. PINK1 is a PTEN (phosphatase and tensin homolog) induced mitochondrial kinase that can be selectively mobilized under mitochondrial stress conditions and lead to the induction of mitophagy [54]. We found an upregulation of PINK1 gene expression in the lower spinal cord in the SNL model, which agrees with a previous study showing that in the SNL model, PINK1 was significantly expressed in the neurons of the spinal dorsal horn, and the expression of PINK1 was increased selectively in GABAergic interneurons [54]. Our findings that both CUR and BDMC reverted SNL-induced PINK1 gene expression in CNS tissues implicate PINK1 gene down-regulation in the anti-nociceptive effects of curcumin in NP.

Dysfunctional mitochondria produce excessive ROS, which over time causes oxidative stress [44]. Our results showed that SOD1 was decreased in the spinal cord, but not in the amygdala, of SNL rats, suggesting that oxidative stress was only elevated at more proximal sites to the injury rather than in remote regions (such as the amygdala). The antioxidant effects of turmeric extract bioactive compounds on oxidative stress depend on their scavenging activity; the scavenging activity of the turmeric bioactive compounds (curcuminoids) is in the following order: curcumin > demethoxycurcumin > BDMC [55]. This would explain why we observed increased SOD1 gene expression only in the SNL+100CUR rats but not in the SNL+50BDMC rats. Our finding that the SNL procedure decreased gene expression levels of Complex I in both the spinal cord and amygdala agrees with a previous study showing that NP caused a significant deficit in Complex I and Complex III-mediated respiration in isolated mitochondria obtained from sciatic nerves [56]. We also showed the upregulation of Complexes I gene expression levels in the spinal cord of SNL rats by both CUR and BDMC treatments, suggesting their (CUR and BDMC) neuroprotective effects in the CNS of animals with NP [56]. Furthermore, our findings showing upregulated TLR4 expression in the spinal cord of SNL rats agree with previous studies showing the activation of TLR4, and consequently the NF-κB activity in the L5 spinal cord of SNL rats [57,58]. The downstream NF-κB signaling pathway is often activated after TLRs activation with tissue damage, and numerous inflammatory cytokines, such as IL-6, IL-1β, and TNF-α, are accumulated in spinal microglia and astrocytes [57,58]. Here we show for the first time that CUR and BDMC significantly decreased TLR4 gene expression, implicating TLR4 in the antinociceptive effects of turmeric curcuminoids.

It is worth mentioning that advanced glycation end products (AGEs), a product of the non-enzymatic reaction, oxidation, rearrangement, and cross-linking between the active carbonyl groups of reducing sugars and the free amines of amino acids, may play an important role in NP [59]. AGEs have been shown to interact with the receptors of AGEs and result in oxidative stress, inflammation response, and signal pathway activation in the development of neurodegenerative diseases [59]. Curcumin, a polyphenol, has great potential to scavenge ROS, which may capture carbonyl species by suppressing AGEs formation, leading to the mitigation of NP. Future studies are warranted to elucidate the effects of curcumin on AGEs in NP progression.

Limitations of the study include the following: (i) due to the limited amount of available tissues of spinal cord and amygdala, this study was not able to assess protein expression using either Western blot or immunohistochemistry, and (ii) only male rats were used in the present study. Future studies are warranted (i) to conduct additional experiments for protein expression in order to confirm the results of gene expression in both tissues, (ii) to evaluate CUR and BDMC in female rats with NP for any gender differences, and (iii) to employ transcriptomic profiling by RNA-seq and metabolites analyses for the better understanding of how CUR and BDMC affect metabolic changes in animals with NP.

In conclusion, both curcumin C3 complex^®^ and BDMC mitigated SNL-induced mechanical hypersensitivity (i) by suppressing gene expression of IBA-1, MFN1 and PGC1α (amygdala), and CD11b (spinal cord), as well as GFAP, DRP1, PINK1, and TLR4 (spinal cord and amygdala) and (ii) by enhancing gene expression of FIS1 (amygdala) as well as Complex I and SOD1 (spinal cord).

## Figures and Tables

**Table 1:** List of primers for mRNA.

Gene	Forward	Reverse
IBA-1	5′-GAG CTA TGA GCC AGA GCA AGG ATT T-3′	5′-ACT CCA TGT ACT TCG TCT TGA AGG-3′
CD11b	5′-TCC AAC CTG CTG AGG AAG CC-3′	5′-TCG ATC GTG TTG ATG CTA CCG-3′
GFAP	5′-AAT CTC ACA CAG GAC CTC GGC-3′	5′-AGC CAA GGT GGC TTC ATC CG-3′
MFN1	5′-AGC TCG CTG TCA TTG GGG AG-3′	5′-TCC CTC CAC ACT CAG GAA GC-3′
DRP1	5′-ACA ACA GGA GAA GAA AAT GGA GTT G-3′	5′-AGA TGG ATT GGC TCA GGG CT-3′
FIS1	5′-CTG CGG TGC AGG ATG AAA GAC-3′	5′-GGC GTA TTC AAA CTG CGT GCT-3′
PGC1α	5′-CAG GAG CTG GAT GGC TTG GG-3′	5′-GGG CAA AGA GGC TGG TCC T-3′
PINK1	5′-TCG GCC TGT CAG GAG ATC CA-3′	5′-CAT TGC AGC CCT TGC CGA TG-3′
Complex I	5′-GGT TTG TCT ACA TCG GCT TCC-3′	5′-TAC AGA AGC TGG CGA TGC AAA-3′
TLR4	5′-TTG CAT CTG GCT GGG ACT CTG-3′	5′-TTC AGG GGG TTG AAG CTC AGA T-3′
SOD1	5′-AGG GCG TCA TTC ACT TCG AG-3′	5′-ACA TGC CTC TCT TCA TCC GCT-3′
β-actin	5′-ACA ACC TTC TTG CAG CTC CTC C-3′	5′-TGA CCC ATA CCC ACC ATC ACA-3′

Abbreviation: IBA-1, allograft inflammatory factor 1; CD11b, cluster of differentiation molecule 11B; GFAP, glial fibrillary acidic protein; MFN1, mitofusin 1; DRP1, dynamin-related protein 1; FIS1, fission mitochondrial 1; PGC1α, peroxisome proliferative activated receptor alpha; PINK1, PTEN-induced kinase 1; Complex I; TLR4, Toll-like receptor 4; SOD1, superoxide dismutase 1.

## Data Availability

Data will be shared upon request.
